# Modern Radiotherapy Concepts and the Impact of Radiation on Immune Activation

**DOI:** 10.3389/fonc.2016.00141

**Published:** 2016-06-20

**Authors:** Lisa Deloch, Anja Derer, Josefin Hartmann, Benjamin Frey, Rainer Fietkau, Udo S. Gaipl

**Affiliations:** ^1^Department of Radiation Oncology, Universitätsklinikum Erlangen, Friedrich-Alexander-Universität Erlangen-Nürnberg, Erlangen, Germany

**Keywords:** radiotherapy, norm- and hypofractionation, SABR, abscopal effect, anti-tumor immunity

## Abstract

Even though there is extensive research carried out in radiation oncology, most of the clinical studies focus on the effects of radiation on the local tumor tissue and deal with normal tissue side effects. The influence of dose fractionation and timing particularly with regard to immune activation is not satisfactorily investigated so far. This review, therefore, summarizes current knowledge on concepts of modern radiotherapy (RT) and evaluates the potential of RT for immune activation. Focus is set on radiation-induced forms of tumor cell death and consecutively the immunogenicity of the tumor cells. The so-called non-targeted, abscopal effects can contribute to anti-tumor responses in a specific and systemic manner and possess the ability to target relapsing tumor cells as well as metastases. The impact of distinct RT concepts on immune activation is outlined and pre-clinical evidence and clinical observations on RT-induced immunity will be discussed. Knowledge on the radiosensitivity of immune cells as well as clinical evidence for enhanced immunity after RT will be considered. While stereotactic ablative body radiotherapy seem to have a beneficial outcome over classical RT fractionation in pre-clinical animal models, *in vitro* model systems suggest an advantage for classical fractionated RT for immune activation. Furthermore, the optimal approach may differ based on the tumor site and/or genetic signature. These facts highlight that clinical trials are urgently needed to identify whether high-dose RT is superior to induce anti-tumor immune responses compared to classical fractionated RT and in particular how the outcome is when RT is combined with immunotherapy in selected tumor entities.

The primary goal of radiotherapy (RT) in cancer therapy is to eliminate the disease by restricting the reproductive potential of tumor cells. This is achieved by the induction of tumor cell death as well as the inhibition of the proliferating capacity of the cells (1). RT is a valuable therapy that is able to control tumor growth, eliminate the tumor, reduce the risk of cancer recurrence, and ultimately to improve survival (2). Radiation predominantly induces DNA damage in the cells (3) and some of its most prominent consequences are apoptosis, necrosis, mitotic catastrophe (MC), autophagy, cell cycle arrest, and/or senescence (Figure 1). About 60% of patients with solid tumors receive RT [15% RT monotherapy, 45% radiochemotherapy (RCT)], making it the most common treatment option for cancer (4, 5). Recent advances in radiation technologies have opened the field for new and promising radiation strategies, such as the stereotactic ablative body radiotherapy (SABR). However, while it has become generally accepted that RT is capable of inducing anti-tumor immunity (6), little is known about the effects of particular high-dose RT on the immune system. In this review, we will, therefore, deal with radiation-induced cell responses, current state-of-the-art radiation protocols, as well as the direct or indirect impact of fractionation schemes and radiation doses on the cells of the immune system, including limitations and draw-backs of today’s radiation research.

**Figure 1:**
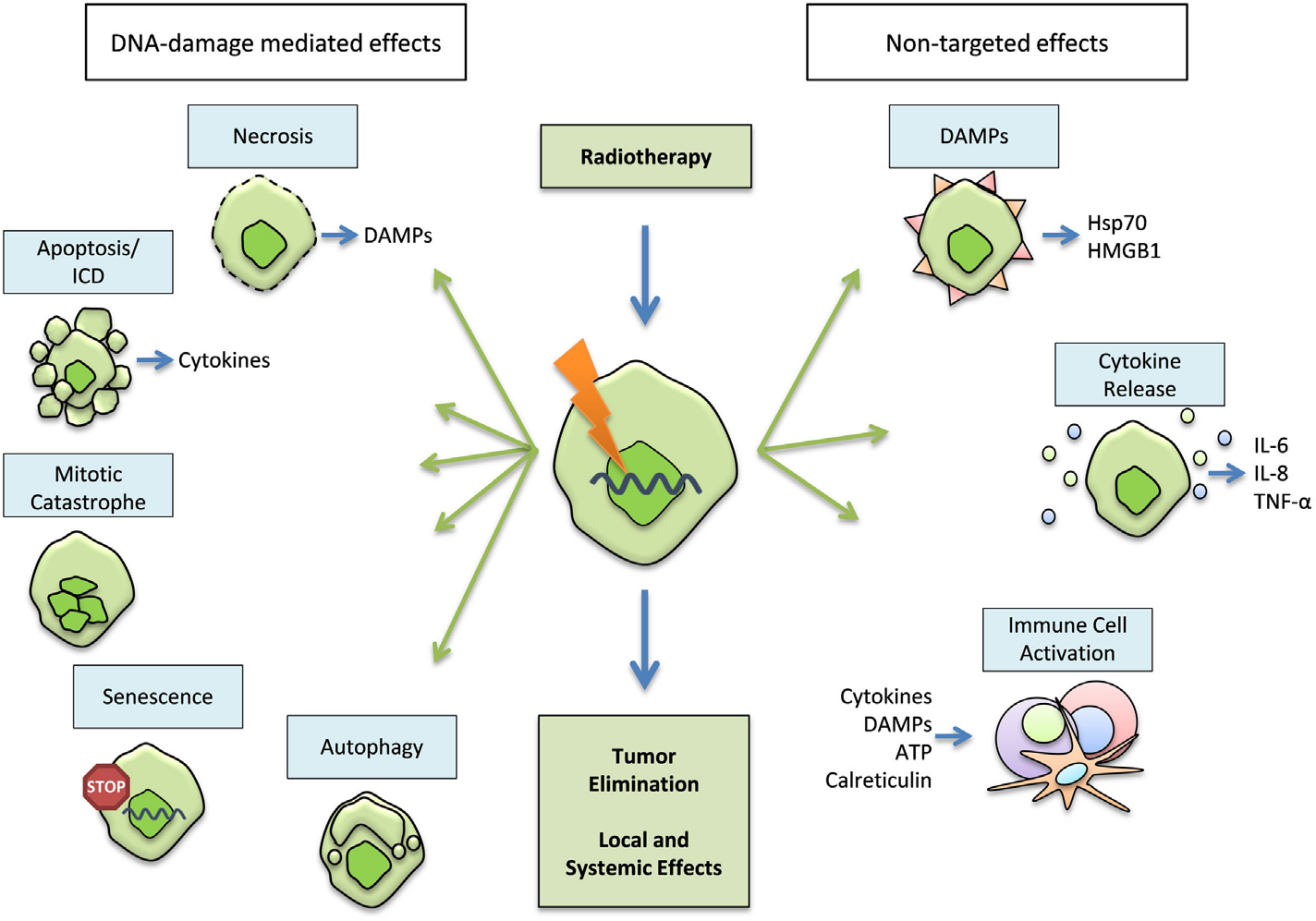
**Primary and secondary effects of radiation**. The primary target of radiation within the tumor cells is the DNA. It aims to eliminate the tumor through inhibition of its proliferating capacity and by induction of cell death. Necrosis, apoptosis, mitotic catastrophe (MC), autophagy, and senescence might occur after radiation-induced DNA-damage. However, radiotherapy (RT) also has a secondary, non-targeted effect that is achieved through a modification of the tumor phenotype, the tumor microenvironment, and/or the induction of an immunogenic cell death (ICD), characterized by the release of danger-associated molecular patterns (DAMPs) and cytokines (e.g., but not exclusively Hsp70, HMGB1, IL-6, IL-8; TNF-α). All of these contribute to the activation of immune-mediated local and distant reactions on the tumor and metastases.

## Radiation-Induced Cell Responses

The main purpose of RT is the induction of directly targeted effects, which are usually well understood and characterized. However, less is known about the so-called non-targeted effects that are often mediated by the immune system and originate from radiation-induced cell death forms.

### Radiation-Induced Apoptosis

Apoptosis is the best characterized form of programed cell death, which plays a major role during cell development, growth, and differentiation, and is important to maintain a healthy homeostatic balance (7, 8). It is characterized by morphological hallmarks, such as cellular shrinkage, chromatin condensation, nuclear fragmentation, and membrane blebbing (9). Cells undergoing apoptosis are engulfed by phagocytes in an anti-inflammatory manner (10, 11). During cancer development, however, tumor cells acquire several resistance mechanisms against apoptosis, such as the expression of anti-apoptotic proteins, the inactivation of pro-apoptotic genes, modifications of the p53 pathway, and an altered survival signaling (12). Following ionizing radiation, an upregulation of various proteins of the death receptor pathway (p53-dependently and -independently) can be observed that might contribute to radiation-induced apoptosis (9). Although apoptosis does not seem to be the predominant form of cell death that is induced by RT in treatment of solid cancer, a positive correlation between tumor response and the amount of spontaneous/radiation-induced apoptosis can be found (13).

### Radiation-Induced Necrosis

Necrosis is often described as an uncontrolled form of cell death, which is morphologically characterized by the gain of volume, swelling of organelles, plasma membrane rupture, and loss of intracellular contents. However, there is emerging evidence that necrosis, such as apoptosis, can be regulated through a set of signal transduction pathways and catabolic mechanisms (14). As cells release damage-associated molecular patterns (DAMPS), such as heat shock proteins (HSP) or high mobility group box 1 (HMGB1) when they undergo necrosis, it is widely considered as an pro-inflammatory and immune activating form of cell death (15, 16). After RT, necrosis often follows MC, finally resulting in local inflammation (3).

### Radiation-Induced Mitotic Catastrophe

Mitotic catastrophe is among the most frequent forms of cell death following irradiation; it occurs after a premature, faulty entrance into mitosis and is characterized as an aberrant nuclear morphology, often resulting in the generation of aneuploid and polyploid cell progeny that almost always die (1, 3, 17). It utilizes anti-proliferative actions, such as apoptosis, necrosis, and senescence in order to stop proliferation of mitotic defective cells. However, as it is functioning as an onco-suppressive mechanism, its failure can also promote unrestricted growth of mitotic defective cells, making it a major possible contributor to tumor development (18). There are two proposed mechanisms for induction of MC: its occurrence as a consequence of DNA damage and deficient cell cycle checkpoints, and a hyper amplification of centrosomes. As most tumor cells have compromised cell cycle checkpoints, e.g., as a frequent consequence from mutated or inactivated p53, alongside altered apoptotic signaling pathways, radiation-induced DNA damage often leads to the induction of MC (1, 17).

### Radiation-Induced Senescence

Reproductive senescence is defined as a condition of permanent cell cycle arrest after cells have reached their proliferative capacity. *In vitro*, cells take on an enlarged and flattened morphology, increased granularity and a vacuole-riche cytoplasm. However, senescent cells are still viable and metabolically active. Today, there are a number of biomarkers typically associated with senescence, some of which can easily be detected via histochemical staining procedures (1, 19). Following RT, replicative senescence is typically observable as a permanent DNA damage response. While replicative senescence is usually initiated when telomeres become critically short and/or the telomere cap is compromised, with both ultimately resulting in exposed chromosome ends, this state can also occur in the case of DNA double-strand breaks (19).

### Radiation-Induced Autophagy

Autophagy is another form of cellular stress response as well as a regulatory way to handle damaged or aged organelles in the cell. Whereas there are different forms of it (such as macro-autophagy, micro-autophagy, and chaperone-mediated autophagy), in general, damaged organelles are enclosed within double membrane vesicles (autophagosomes) that consequently fuse with lysosomes and are ultimately digested from the lysosomal proteases (20). Thus, autophagy usually acts in a pro-survival manner that helps to sustain cellular homeostasis and genomic integrity. Within tumors, the rate of autophagy is often upregulated in comparison to healthy tissues (21). Tumor cell autophagy is thought to have different and contrasting functions. One of it is a cytoprotective one, possibly serving as a protective measure to protect the tumor against therapy-induced apoptosis (21, 22). The other one is a rather cytotoxic function that might function to promote tumor cell killing, but is rarely observed in conventional treatments (22). While, theoretically, both forms of autophagy in tumor cells might be subject to alterations in order to further enhance the effectiveness of treatment modalities, the role of autophagy in cancer is still controversial and is thus, subject to extensive research (21, 22): even though it has already become obvious that DNA damage responses, inflammation and autophagy are inter-connected (23, 24) and it seems that RT almost uniformly promotes autophagy in tumor cells, it is currently unsure whether RT-induced autophagy should be promoted or inhibited for a more beneficial outcome (25).

### Non-Targeted Effects of Radiation

However, next to these primary effects of RT, one can also observe secondary effects following radiation (26): the activation of the immune system via the induction of immunogenic cell death (ICD) by RT (27) (Figure 1). Besides the main target, the induction of DNA damage, ionizing radiation is also able to modify tumor phenotypes as well as the tumor microenvironment. These, the so-called non-targeted effects, can contribute to anti-tumor responses in a specific and systemic manner and possess the ability to target relapsing tumor cells as well as distant metastases (28). In order to enhance RT-induced anti-tumor immunity, additive chemotherapy (CT) and immunotherapy (IT) have proven to be useful tools. However, there are still many open questions and various hypotheses regarding the effects of different concepts of RT on immune activation. One of them, the question if today’s RT schemes have heterogeneous influences on the immune system, and if certain fractionation schemes and doses could be advantageous over others, will be discussed here.

## History of Radiotherapy

In order to better understand some of today’s treatment prospects, a short summary on the rather rapid development of clinical RT is helpful. We, therefore, will focus on the history of RT within the next paragraph: just a few weeks after Wilhelm C. Röntgen discovered X-rays in 1895, Grubbé treated an advanced ulcerated breast cancer in Chicago in 1896 using X-rays (29). Complications and negative side effects of early X-ray applications were discovered quickly and taken into account (30). In that matter, Thor Stenbeck used smaller doses of radiation over a longer period of time to treat skin cancer in Stockholm (31). In 1906, Bergonié and Tribondeau were experimenting with X-rays and rat testicles in order to investigate selective influences of X-rays on healthy tissues. They discovered that X-rays are most effective on cells having (a) a high proliferative rate, (b) a long life-span with many divisions, and are (c) unspecialized (32). Within the first 40 years after Röntgen’s discovery, RT became a routinely used clinical application for both malignant and inflammatory diseases. In the 1920s, animal sterilization experiments in rams, carried out by Regaud, showed the advantages of splitting the administered radiation into smaller daily fractions: this way sterilization of the ram testes was able to be carried out with minimal necrosis to the scrotum (33). In 1934, Coutard published a paper on principals of X-ray therapy on malignant diseases where he showed an impact of treatment time and dose on both, the cancer and the surrounding tissues (34). This can be seen as the founding of the time–dose factor concept. As a result of these and other radiobiological experiments, a consensus on a fractionated treatment scheme was found (31). While Coutard shed light on time–dose dependencies, Baclesse helped to gain insight on dose–volume relationships (31, 35). In the 1960s, Ellis and colleagues investigated biological effects with regard to dose–time fractionated factors (31, 36).

Over time, further developments lead to more and more precise linear accelerators that paved the way for today’s advanced treatment strategies allowing the administration of higher tumor doses while sparing healthy tissues. Specialized irradiation devices, multi leaf collimators, treatment planning software, and new treatment methods, such as intensity modulation and dynamic beam shaping, allow a high-precision tumor irradiation. However, a great part of today’s radiation schemes are still based on the data that were collected in the very beginnings of clinical RT.

## Advancements and State-of-the-Art Radiotherapy

### Considerations About Radiotherapy

Despite modern developments in RT, treatments always affect surrounding healthy tissue, at least to a certain degree. Thus, it is important to be aware of general side effects and to define organs at risk (OARs). However, modern clinical advances provide new opportunities, enhancing the anti-tumor effect of RT, while simultaneously lowering the damage of adjacent cells and thereby reducing possible negative side effects (5). The most common state-of-the-art RT options will be introduced in the following.

### Conformal Radiotherapy

In the past, 2D open-field irradiation was applied without taking a sparing of healthy tissue and especially possible OARs into account. Today, however, all radiation therapy treatments are prepared with a 3D treatment planning system and are performed with the help of a treatment device, which is combined with a reproducible positioning system and, thus, allows the shaping of irradiation beams around the tumor volume. In this way, the tumor region can be irradiated, while healthy tissue can be spared.

### Intensity Modulation Radiotherapy

Intensity-modulated radiotherapy (IMRT) planning is carried out opposed to the planning order in conformal radiotherapy. Meaning that all beam dose distributions are derived from the target dose distribution (37). This is done via multi leaf collimators that allow applying a very high dose on the tumor, while decreasing irradiation of healthy tissue to a minimum. The beam intensity is modified heterogeneously for each irradiation field. In that way, different doses can be given across the tumor, while U-shaped dose distributions allow avoiding OARs, which in turn considerably reduces the risk of long-term side effects.

### Volumetric Modulated Arc Therapy

The volumetric modulated arc therapy (VMAT) aims for beam intensity distribution by changing multi leaf collimators configurations and an additional dose rate variation. During treatment, the gantry turns for a maximum of one single rotation (38). That way, irradiation times can be reduced (39) to 1.5–3 min for a 200 cGy fraction (38).

### Image-Guided Radiotherapy

With image-guided radiotherapy (IGRT) the precision of irradiation can be further increased. IGRT involves both the reduction of positioning errors during treatment, and precise segmentation and evaluation of the clinical tumor volume (40). In order to achieve this, additional scans can be made to evaluate size, localization, and shape of the tumor that can be checked against the digital reconstructed radiographs of the planning CT. This can be done either before or during the entire RT procedure, with the latter constituting for a more elaborate process. Thus, this technique promotes a very precise tumor targeting and consequently a better local control and enhanced chances of recovery, while the risk of unwanted side effects is reduced. A special type of IGRT is the four-dimensional, adaptive RT, whereby the fourth dimension is time. The advantage of this method is the adjustment of position changes during treatment, which is, e.g., necessary in lung cancer where the tumor moves by regular breathing.

### Stereotactic Body Radiotherapy and Radiosurgery

All these advancements paved the way for more and more precise RT set-ups that also allow the administration of a single, high, very accurately targeted irradiation dose (stereotactic radiosurgery) or fractions of larger doses (stereotactic RT). Both allow for a very precise radiation strategy by using image-guided and computer-assisted systems. Currently, radiosurgery is used for some types of brain cancer and in clinical trials for other entities, such as prostate cancer. Irradiation is applied from several positions around the body, resulting in high irradiation doses delivered on the tumor while spreading only relatively low doses on surrounding, healthy tissue, thus, further reducing the risk of possible side effects. This technique is especially relevant for brain cancer as well as for tumors that are clearly distinct from the surrounding tissue in prostate, lung, spine, liver, pancreas, or kidney (41).

### Radiation Dose Application

In general, fractionation schemes are based on year long experience as well as on positive therapy outcome and, thus, can vary greatly. While a high number of patients receive classical or conventional RT with 1.8–2.0 Gy per fraction, choosing alternative fractionation protocols can be advantageous in order to achieve a better tumor control with fewer toxicities (42). Today, there are many RT strategies and the total dose can be administered either in the form of a few high doses, or in smaller fractions over a longer period of time depending on tumor entity, placement, and therapeutic goals. Generally spoken, fractionation has the advantage of helping the surrounding, healthy, slower proliferating tissue to recover, while fast proliferating tumor cells can accumulate a greater amount of DNA damage in various, more or less radiosensitive, phases of the cell cycle. However, fractionation protocols also have diverse effects on the tumor, the surrounding tissue, and thus on the immune system.

In most cases, RT is administered in the form of conventional fractionation where multiple fractions of 1.8–2.0 Gy/day are administered five times a week over 3–7 weeks (4). In a hyperfractionated therapy setting, conventional doses are brought down into smaller doses without a change in the overall treatment duration. This way the therapeutic potential between late responding normal tissue and tumor tissue is increased (43). Typically, patients receive 0.5–2.0 Gy/fraction with two fractions/day and two to five times a week, over a time period of 2–4 weeks. Patients treated with a hypofractionated scheme receive doses of 3–20 Gy/fraction with one fraction/day resulting in a reduced therapy time. However, there are also other forms of fractionation protocols, for example accelerated protocols: here, tumor growth during the treatment is minimized because of a shortened overall treatment duration while still using conventional doses (43). Another variant is the so-called accelerated hyperfractionation (1.0–1.6 Gy/fraction in five and more fractions/week). However, the limitations in this protocol are mainly due to acute toxicities, as both strategies independently increase acute reactions (43). In the case of SABR, small lesions, mainly in the brain, are treated with higher doses in fewer fractions resulting in a promising therapeutic outcome. Fractions and doses used for SABR usually are within a 8–30 Gy window and consist of 1–5 fractions (44).

In most fractionation schemes, the set values differ greatly, depending on the treated cancer entity but also on the clinic or treatment facility. However, from a biological point of view, these deviations might influence the immune system and tumor cell responses differently and lead to a diversified treatment outcome.

## Challenges of Current Radiobiological Research

Even though there is extensive research carried out in the field of radiation oncology, most of the clinical studies only consider effects of radiation on the local tumor tissue. The influence of dose fractionation and timing particularly with regard to immune activation is not satisfactorily investigated so far. However, this is of particular interest, since recent studies, including additive immune therapy approaches, showed that not every therapy combination of classical RT concepts and IT is equally successful (45).

Another compounding issue is the comparability of pre-clinical models with patient treatment: as the transmission of *ex vivo* and *in vivo* studies onto clinical trials and the outcome in patients is already a widely discussed subject, it is even more important that experiments are set-up in a way that follows clinical radiation schemes. However, in some cases that also means that doses and numbers of fractions need to be adjusted in order to retain the biological effective dose. This is also a reason for complications in transferability of models as single doses in mice might differ in their effect on the experimental tumor from those in humans. Furthermore, not all studies even meet these criteria, and dose and fraction sizes are often chosen to specifically meet the needs of the used model, or according to the possibilities of the facility carrying out the experiments, thus, resulting in a hampered comparability. Therefore, the rationale for the chosen dose and fractionation should be included in every publication with pre-clinical model systems.

### Biologically Effective Dose

The biologically effective dose (BED) is used for isoeffective dose calculations. It is defined as a measure to determine the biological dose delivered via a combination of dose per fraction and a total dose to the precise tissue that can be characterized by its α/β ratio. Treatment doses differ in administration, dose per fraction and total dose of the irradiation. Conversion follows the linear-quadratic model, first described by Douglas and Fowler (46), which characterizes the cell survival curve of both tumor and healthy tissue. Thus, the BED can be used as an approximate measure to adjust fraction size for a wide range of dose fractions (47) and to quantify treatment expectations (48). In order for a more practical approach for the clinic, the BED can be converted into the biological equivalent dose that is calculated in 2 Gy per fraction (EQD2). To better compare doses used in pre-clinical and clinical settings, information on the BED or EQD2 should be mandatory (47).

## Influence of Distinct RT Concepts on Immune Activation

The establishment of solid tumor tissue presumes that the tumor cells have successfully evaded immunosurveillance and are still able to do so for longer periods of time. Usually, tumor cells can be eliminated by the immune system through a collaboration of the innate and adaptive immune system that effectively detects and destroys tumor cells. It is, however, possible that single cells are not eliminated during the process; those cells can progress into an equilibrium phase: in this state the immune system is still able to keep the transformed cells under control. However, in the final stage of tumor escape, one of the hallmarks of cancer, the dormant tumor cells outgrow the surveillance of the immune system due to their reduced immunogenicity, establish a immunosuppressive microenvironment, and begin to grow progressively (49). Even though this involvement of the immune system has been known for a long time, it was generally believed that there are no direct synergies in between RT-induced local tumor responses and the immune system. This is due to the immunosuppressive properties of RT, as lymphocytes are known to be radiosensitive and their levels in the peripheral blood are lowered after RT (50). The same effect can be observed in the bone marrow, where RT has a damaging effect on monocyte and granulocyte precursors, as well as on natural killer cells (51).

Nevertheless, there are emerging hints that the immune system can also be stimulated by RT (52) and today it is accepted that, next to radiation-induced cell death and growth inhibition, radiation can also activate the immune system (52–54). These systemic and immune-mediated effects of RT have been described as abscopal effects of RT (55, 56).

Currently, the influences of present RT concepts on the immune system are still only fragmentarily understood and it would be beneficial to gain a better insight about the effects of week-long RT on a molecular, cellular, and tissue level (4) as well as its effects on immune cells (53). While classical radiobiology has created a general understanding about survival curves based on varying radiation doses and treatment volumes, and extensive research concerning DNA damage and repair capacities following RT has been carried out, there is still a lack of data in pre-clinical and clinical studies with regard to radiation and its effects on the immune system (51, 57, 58). Consequently, data about the impact of RT concepts on immunological consequences are scarce and not conclusive (57), and present knowledge will be reviewed in the following.

### Radiosensitivity of Immune Cells

In general, lymphocytes are among the most radiosensitive cells within the body (50, 59). Next to the induction of cell death (50), the underlying mechanisms of the immune-suppressive effect of RT are thought to be the inhibition of the antigen-expressing function as well as the downregulation of co-stimulatory molecules, such as CD80 and CD86 on immature DCs (60). Furthermore, an altered cytokine profile, and RT-induced proliferation stop of their progenitor cells contribute to it (59). On the other hand, RT is also able to stimulate the expression of immunomodulatory molecules such as co-stimulatory molecules in antigen-presenting cells (APCs), T cells, and stromal cells (61) as well as to modify the function of DCs in a way that constrains endogenous antigen presentation while increasing their cross-presentation abilities (62).

However, while lymphocyte radiosensitivity is well known, the effects of distinct RT doses or administrations on immune modulation and on different immune cell subsets are still not fully understood (63). Falcke et al. are currently investigating the effects of ionizing radiation on different immune cells subsets, with special regard to their individual radiosensitivity. They found that T cells, B cells, and natural killer cells are among the most radiosensitive immune cells, while monocytes are much less sensitive (64). Kaina and colleagues revealed that macrophages are even more radioresistant than monocytes (65) with doses of ionizing radiation up to 2 Gy having no impact on viability and functionality of activated macrophages (66).

Merrick et al. performed experiments with regard to the radiosensitivity of DCs. They found that DCs were more resistant against radiation-induced (up to 30 Gy) apoptosis than expected, with only small changes in their surface phenotype alongside with their endocytic, phagocytic, and migratory abilities (67). However, irradiated DCs had reduced effectiveness in mixed lymphocyte reaction experiments compared to non-irradiated ones. Furthermore, matured DCs produced less interleukin (IL)-12 than the controls while IL-10 levels remained stable. They, thus, hypothesize that IR has an effect on DC function possibly leading to a shift in the DC-mediated balance between T-cell activation and toleration.

With regard to T cells, ablative RT increases T cell priming in lymphoid tissues, possibly contributing to the extermination of the primary tumor as well as distant metastases in a CD8^+^ T cell-dependent manner (63). It has also been shown that high doses of ablative RT given in as less as one to three fractions are able to generate adaptive immune responses that result in a regression of the tumor (68). In the case of myeloid cells, RT can also have various effects, as summarized in Ref. (69). In most cases, RT-associated recruitment of myeloid-derived suppressor cells (MDSCs) and M2 tumor-associated macrophages has a negative effect by mediating immune evasion and tumor growth (69). In some cases, however, this effect can be reversed. In that matter, Deng et al. (68) showed that a combined RT and anti-PD-L1 therapy is able to reduce the numbers of MDSCs and, thus, their suppressive effects on the immune system.

These and other examples show that, next to the immunosuppressive properties of RT on immune cells, radiation can also cause a reduced tumor growth outside the irradiated field. This effect is an immune-mediated effect that is observable after the administration of various radiation doses and fractionation schemes, but mostly only in combination with additional immune stimulation (58). Furthermore, more knowledge should be gained on the genetic landscape of primary vs. metastatic and recurrent tumors as it has just recently been performed by whole-exome sequencing of head and neck squamous cell carcinoma (HNSCC) (70). Also, tumors with high somatic mutation prevalence do respond better to immunotherapies (71). RCT in multimodal settings might generate more neo-antigens that are the origin for initiating anti-tumor immune responses. Coupling radio-immunotherapies with agents that impact on DNA repair pathways might be particularly constructive in this respect (72). It is important to stress that more clinical trials are needed to more clearly delineate the best combination of RT with IT and/or DNA repair pathway inhibition and, as outlined above, that the optimal approach may differ based on the tumor site and/or genetic signature.

Taken all this information into account, it is not exaggerated to formulate that the immune system plays a vital role in the fight against cancer and that there is a lack of pre-clinical and clinical trials focusing in particular on the role and functionality of immune cells after RT.

## Radiation and Immunogenic Tumor Cell Death

Ionizing radiation can render the tumor microenvironment more immunogenic. Following RT, stressed and dying cancer cells release a variety of substances, including reactive oxygen as well as nitrogen species alongside with cytokines, such as IL-6, -8, and tumor necrosis factor-α (TNF-α) that are all able to stimulate the immune system and to promote local bystander effects. RT-induced apoptosis is characterized by the exposure of ER-derived proteins, such as calreticulin and by the release of DAMPs. This leads to the recruitment of APCs and, thus, the adaptive immune system resulting in a distant out of field or abscopal effect. This form of apoptosis is also known as ICD (73). Necrotic cells also release the so-called danger signals, such as Hsp70 or HMGB1. These bind to receptors, e.g., to toll-like receptors (TLRs) on APCs such as DCs, induce their maturation and promote cross-presentation of tumor antigens (53, 74). Another inflammatory molecule associated with immunogenic cancer cell death is adenosine triphosphate (ATP). By binding to receptors on DCs, ATP can stimulate the release of IL-1β that in turn can promote T cell priming (74). However, in many cases tumor cells do not directly die after treatment. But also, in this case, sole exposure of calreticulin might render the tumor more sensitive to killing by cytotoxic T lymphocytes (75, 76). An additional effect of ionizing radiation is the normalization of tumor vasculature that can also inhibit tumor growth (73), moreover, these effects go beyond direct nuclear damage (77). As with many of the described effects, radiation-induced changes in tumor vasculature are also highly dependent on the applied doses and radiation schemes (78). However, even though there is a good understanding of these effects, surprisingly little is known about how ionizing radiation and RT-connected responses of the immune system alter the tumor microenvironment (68, 77) with special regard to the impact of different radiation regiments.

### Impact of Distinct Radiation Doses and Fractionation Schemes on Tumor Cell’s Immunogenicity

Rubner et al. showed in an *in vitro* model system that fractionated RT given at 5 × 2 Gy is the main stimulus for the induction of an ICD in glioblastoma cell lines (79). Furthermore, fractionated radiation with 2 Gy as single dose induced the release of Hsp70 in p53 mutated and O6-methylguanine methyltransferase negative glioblastoma cell lines. As the danger signal Hsp70 is able to activate DCs, classical fractionation might be beneficial to create a favorable tumor microenvironment for the integration of IT. An experimental study carried out by Kulzer et al. dealt with the capability of the colorectal tumor cell line SW480 to activate DCs after the application of various RT schemes (80). Immature DCs cultivated with supernatants from tumor cells treated with either classical (5 × 2 Gy) or hypofractionated (3 × 5 Gy) RT secreted significantly elevated levels of the immune activating cytokines IL-12p70, IL-8, IL-6, and TNF-α compared to immature DCs stimulated with supernatants of SW480 cells treated with 1 × 15 Gy. Furthermore, only supernatants of fractionated irradiated cells resulted in elevated DC-maturation markers after immature DC co-cultivation and merely those were able to stimulate CD4^+^ T cells in an antigen-specific matter. Tsai et al. investigated gene expressions in the breast, prostate, and glioma cell lines MCF-7, DU145, and SF539 after RT with either 1 × 10 Gy or 5 × 2 Gy (81). They found differences in gene expression patterns depending on radiation protocols. In that matter, a fractionated radiation resulted in a more robust gene induction than that of single doses. In addition, they also found a small subset of *interferon* (*IFN*)-related identical genes, a group of genes that has been implicated in inflammation, upregulated in all three tumor cell lines following fractionated irradiation. Deng et al. showed that in DCs the adaptor protein STING was required for IFN-γ induction in response to irradiated-tumor cells (82). Here, a high single ablative dose of 20 Gy was used for the experiments.

**Figure 2:**
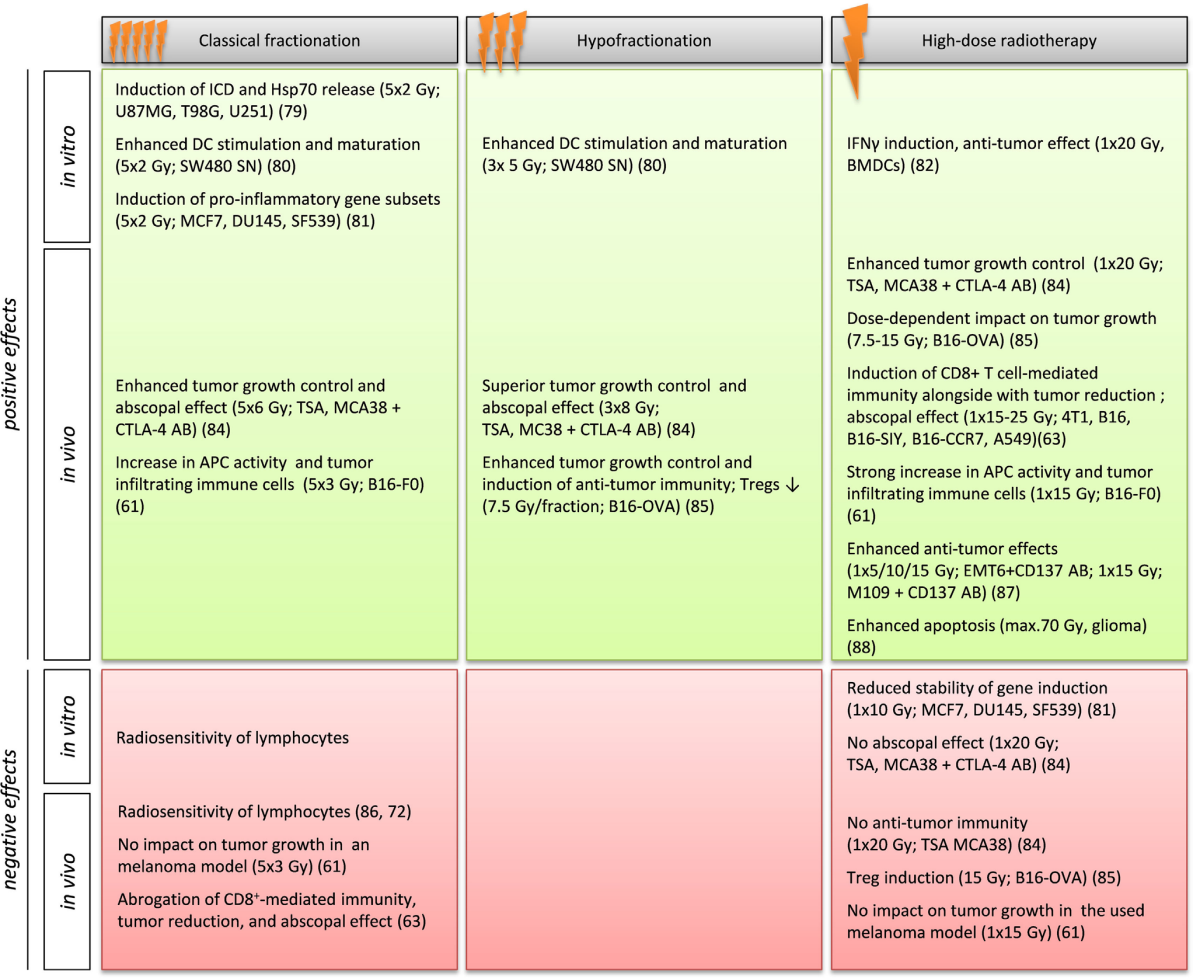
**Influence of RT schemes on the tumor cells immunogenicity in selected *in vitro* and *in vivo* model systems**. Gy, Gray; ICD, immunogenic cell death; Hsp70, heat shock protein 70; DC, dendritic cell; SN, supernatant; APC, antigen-presenting cell; Treg, regulatory T cell; IFNγ, interferon γ; CTLA-4, cytotoxic T lymphocyte-associated protein 4; AB, antibody.

Taken together, these selected *in vitro* results propose (1) the ability of ionizing radiation to induce immunogenic effects and (2) a superior outcome of fractionation schemes over single high doses of radiation (Figure 2). However, these *ex vivo* assay systems only give hints about the immunogenic potential of the tumor cells and pre-clinical *in vivo* experiments and clinical studies are ultimately needed (83).

Pre-clinical mouse models often suggest a more beneficial outcome if higher doses are used in hypofractionated schemes (84, 85). Multhoff et al., thus, support the assumption that long-lasting, daily repeating RT leads to lymphocyte death (53) and longer breaks during the radiation are needed to give the immune system time to act and re-act (86). Dewan et al. report that fractionated, but not single doses induce immune-mediated abscopal effects in combination with a CTLA-4 antibody (84). While a single high dose (1 × 20 Gy) and fractionation regiments both had the ability to control primary tumor growth in a TSA breast cancer and MCA38 colon cancer mouse model, no effects on a distant secondary tumor could be found after ablative RT. However, they also found that 3 × 8 Gy was superior to lower doses of RT that have been applied more often (5 × 6 Gy), suggesting advantageous effects of hypofractionated schemes. Schaue et al. carried out experiments in mice bearing B16-OVA melanoma. They found that single doses of radiation from 7.5 to 15 Gy had a dose-dependent impact on tumor control, and tumor-reactive T cells, with an offset at 15 Gy. However, after 15 Gy they also found increasing numbers of Tregs. A single dose of 5 Gy had only little impact on tumor control. However, a fractionated treatment scheme with medium-sized doses of 7.5 Gy/fraction resulted in the best tumor control and immunity with low Treg numbers (85). By contrast, Lee et al. observed a CD8^+^ T cell-dependent immunity and tumor reduction, together with a reduced relapse of the primary tumor, as well as an eradication of metastases in some cases particular after high dose or ablative RT with 1 × 15–25 Gy in various cancer models. They also show that these ablative RT-mediated effects are abrogated by conventional fractionated RT or adjuvant CT while locally administered IT enhances the effects (63). Thus, they imply a limitation rather than a stimulation of RT-mediated tumor immunity caused by some of the currently used RT/CT strategies. Lugade et al. evaluated anti-tumor immune responses after single dose (1 × 15 Gy on day 7) or fractionated (5 × 3 Gy on days 7–11) RT in mice injected with OVA-expressing B16-F0 tumors (61). With regard to long-term tumor control, neither of the two RT schemes was able to effectively prevent tumor growth. Non-irradiated mice showed large tumors by day 14 and single dose treatment was rather effective during the initial time period of 14 days. However, at day 30, the mice that had received a single dose of 15 Gy also had to be sacrificed due to a large tumor burden. Surprisingly, the 5 × 3 Gy fractionated scheme showed only slightly better tumor growth control rates than non-irradiated controls. With regard to APC activity, the single high-dose treatment resulted in a threefold increase in APC activity, while mice treated with fractionated RT showed APC activity to a lesser extent. Using immunohistochemistry and flow cytometry analyses, numbers of total immune cells (defined by CD45 expression) and immune cell subtypes infiltrating the tumor were also analyzed. In general, irradiation resulted in higher numbers of total CD54^+^ cells and for all of the subsets than in non-irradiated tumors. With regard to RT schemes, in mice that received the single high dose the average number of infiltrating immune cells per tumor mass was higher than in those receiving the fractionated scheme. These results suggest that single high doses represent a more beneficial treatment plan, at least in this model. Shi et al. investigated the effects of an anti-CD137 antibody in combination with different fractionation schemes in murine lung (M109) and breast (EMT6) carcinoma models (87). CD137 is a member of the TNF-receptor superfamily and is able to deliver a co-stimulatory signal for T cell activation. Administration of the antibody in combination with a single dose RT (5, 10, or 15 Gy) resulted in an enhanced anti-tumor effect in the case of EMT6 tumors. Regarding M109 tumors, only the highest dose of 15 Gy resulted in enhanced tumor response rates. These results once more show that an individual evaluation of tumor entity and chosen RT treatment plan are crucial for an optimized outcome. Witham et al. examined if radiosurgery also induces apoptosis next to necrosis in an experimental rat glioma model (88). They aimed to investigate whether radiosurgery (a) induces tumor apoptosis and if this correlates with a survival benefit and (b) whether the extend of apoptosis and its time course provides a basis for other treatment modalities, such as IT. They treated Fischer 344 rats with established intercranial 9L gliosarcomas with radiosurgery (max. dose 70 Gy) and sacrificed the animals at 3, 6, 12, 24, 48, 72 h, and 1, or 2 weeks after treatment. They revealed that tumor apoptosis was significantly higher at the 6-, 24-, and 48-h time point in comparison to untreated controls. They, thus, hypothesize that radiosurgery induces apoptosis in a time-dependent manner, possibly giving the opportunity to combine radiosurgery with IT, that utilizes tumor apoptosis for antigen presentation. Park et al. investigated the influence of the expression of the immune checkpoint molecule PD-1 on the systemic anti-tumor response induced by SABR in pre-clinical melanoma and renal cell carcinoma models (89). They found that the observed abscopal effect was tumor specific and that in particular a combination of SABR with PD-1 blockade was able to induce it. Selected examples of the influence of RT regiments on immune stimulation are summarized in Table 1.

**Table 1:** **The influence of fractionation regiments on immune stimulation in selected pre-clinical *in vitro* and *in vivo* models**.

Experiment type	Tumor entity	Tumor model	Fractionation regiment	Additional therapy	Observed immune modulations	Source
*In vitro*	Glioblastoma	U87MG, T98G, U251	5 × 2 Gy	±TMZ and VPA	Hsp70 and HMGB1 secretion ↑ in irradiated tumor cell lines	(79)
*In vitro*	Colorectal carcinoma	SW480	5 × 2 Gy, 3 × 5 Gy, 1 × 15 Gy	–	5 × 2 Gy and 3 × 5 Gy: IL-12p70, IL-6, IL-8, and TNF-α secretion↑, DC-maturation markers↑, CD4^+^ T cell stimulation with SN treated iDCs	(80)
*In vitro*	Breast, prostate, glioma	MCF-7, DU145, SF539	5 × 2 Gy, 1 × 10 Gy	–	5 × 2 Gy: more robust gene induction, upregulation of *IFN*-related genes	(82)
*In vitro*	Breast cancer	MCF-7, MDA-MB231	4 × 4 Gy, 6 × 3 Gy, 1 × 4/10/20 Gy	HT, ± zVAD-fmk	Clonogenicity MCF-7↓↓, MDA-B231↓ Hypofractionation is main stimulus for cell death induction and DC activation in MDA-MB231 cells	(90)
*In vitro*	Prostate cancer	LNCaP, PC3, DU145	10 × 1 Gy, 1 × 10 Gy	–	10 × 1 Gy: more robust immune response gene induction, number of induced immune genes PC3↑↑, DU145↑, LNCaP↑	(91)
					Induction of pro-inflammatory DAMPs and cytokine modulation (10 × 1 Gy ↑↑, 1 × 10 Gy ↑)	
*In vitro*	Breast cancer	TSA	1 × 2/5/10/20 Gy	Carboplatin, oxaliplatin	Dose-dependent induction of ICD Chemotherapeutic enhancement of ICD	(92)
*In vitro*	C57BL/6J, *Tmem173*−*/*−**, and *Irf3*−*/*−** mice	BMDCs	1 × 20 Gy	–	STING is required for the anti-tumor effect of radiation and type I IFN induction	(82)
*In vitro*	Melanoma	MeIJuSo	1 × 1/4/10/25 Gy	–	RT modulates peptide repertoire and enhances MHC class I expression in a dose-dependent manner	(93)
*In vitro*	Colorectal carcinoma	HCT116, SW620	1 × 10 Gy	–	HCT116: OX40↑, 41BB↑ ligands Induction of genes associated with T cell effector activities	(94)
*In vivo*	Breast carcinoma	TSA, MCA38	5 × 6 Gy, 3 × 8 Gy, 1 × 20 Gy	9H10	Growth reduction of secondary, out-of-field tumors (5 × 6 Gy + 9H10↑, 3 × 8 Gy + 9H10↑↑)	(84)
*In vivo*	Melanoma	B16-OVA	1 × 5/7.5/10/15 Gy	–	Dose-dependent increased tumor control (5 Gy↓) and tumor-reactive T cells (15 Gy↓), 15 Gy Tregs↑, 7.5 Gy superior tumor control and low Treg numbers	(85)
*In vivo*	Mammary and lung carcinoma, melanoma	4T1, B16, B16-SIY, B16-CCR7, A549	1 × 15–25 Gy		CD8^+^ T cell-dependent immunity↑, tumor reduction↑, abrogation of observed effects after conventional fractionated RT or CT, IT enhances the observed effects	(63)
*In vivo*	Melanoma	OVA expressing B16-F0	5 × 3 Gy, 1 × 15 Gy	–	No significant effects on tumor growth, APC activity, total immune cells, tumor infiltrating immune cell subtypes: 5 × 3 Gy↑, 1 × 15 Gy↑↑	(61)
*In vivo*	Lung and breast carcinoma	M109, EMT6	1 × 5/10/15 Gy	Anti-CD137 antibody	EMT06: all doses ↑ anti-tumor effect M109: 15 Gy ↑ anti-tumor effect	(87)
*In vivo*	Rat glioma		70 Gy max dose	–	Tumor apoptosis↑ after RT in a time-dependent manner	(88)
*In vivo*	Breast cancer	TSA	3 × 8 Gy	Imiquimod, cyclophosphamide	Combination of imiquimod, RT, and cyclophosphamide induces protective immunologic memory, tumor infiltration by CD11c^+^↑, CD4^+^↑, CD8^+^↑ cells	(95)
*In vivo*	Colon cancer	CT26, MC38	1 × 30 Gy	–	Transformation of immunosuppressive tumor microenvironment, CD8^+^↑ tumor infiltrates, MDSCs↓	(96)
*In vivo*	Sarcoma	MethA, C3	3–5 × 10 Gy	DC administration	Anti-tumor response↑	(97)
*In vivo*	Melanoma	D5	5 × 8.5 Gy	Intratumoral DC administration	Local and systemic anti-tumor response↑	(98)
*In vivo*	Lung carcinoma, fibrosarcoma	LLC, T241	5 × 10 Gy, 12 × 2 Gy	–	5 × 10 Gy: out of field tumor growth↓	(99)
					12 × 2 Gy: LLC tumor growth↓, implicated dose-dependent efficiency of abscopal effect, p53 as key mediator for the abscopal effect	

*↑, increase; ↓, decrease; Gy, Gray; TMZ, temozolomide; VPA, valproic acid; Hsp70, heat shock protein70; HMGB1, high mobility group box 1; IL, Interleukin; TNF-α, tumor necrosis factor-α; SN, supernatant; DC, dendritic cell; iDC, immature DC; IFN, interferon; BMDC, bone marrow-derived cells; STING, stimulator of IFN genes; HT, hyperthermia; DAMP, danger-associated molecular pattern; ICD, immunogenic cell death; Treg, regulatory T cell; RT, radiotherapy; CT, chemotherapy; IT, immunotherapy; MDSC, myeloid-derived suppressor cell; APC, antigen-presenting cells*.

## Clinical Hints for Immunogenic RT

While fractionation generally reduces toxicity to the surrounding tissue and enhances DNA damage in tumor cells, there is also evidence that prolongation of the overall treatment time has a negative impact on the outcome of certain tumors (100). In that matter, there is a possibility to alter fractionation protocols in an accelerated manner, however, this is not suitable for all cancer entities, as, e.g., OARs might be close by. Innovative RT protocols have to consider this as well as to take the immunogenic potential of distinct doses and fractionations and of the genetic signature of the tumor into consideration. Also, there are no consistent radiation schemes throughout different centers. However, there are ambitions to come to a conclusion regarding detailed recommendations of the treatment process. In that matter, Guckenberger et al. have given a detailed report about guiding principles for the treatment of stage I non-small cell lung cancer (NSCLC) (101). Sterzing et al. gave a detailed overview of current SABR treatment in the case of liver tumors (102).

### Clinical Evidence for Enhanced Immunity after RT

Next to pre-clinical evidences that demonstrate how RT can modulate the immune system and enhance immunity, there is also clinical evidence for this phenomenon. The *abscopal effect*, as already described by Nobler in 1969 (103), defines the regression of distant tumors outside of the irradiation field without the help of additional therapeutic options. As nowadays we have prove that this effect is immune mediated (56, 86), the description *systemic immune-mediated effects of RT* is more appropriate and, thus, should be used. Even though there are only few reported cases, this effect clearly shows the ability of RT to modulate the immune system and cases for various cancer entities have been described (104):

In that matter, Konoeda reported an abscopal effect in pre-operatively irradiated patients, which was verified by palpation on metastatic lymph nodes in 15 out of 42 cases (35.7%), and 22 out of 42 cases (52.4%) through histopathological findings (105). He also reports a significantly higher incidence in patients under 55 years of age (age distribution: 29–84 years; mean 54 years) and in those who showed infiltrating lymphocytes allocated around the degenerated cancer cells within the irradiated primary tumors, further supporting the involvement and stimulation of the immune system after RT. Using monoclonal antibodies in immunohistological stainings, the infiltrating lymphocytes were identified as CD8^+^ and CD4^+^ T cells. He, thus, concluded that the observed effect was caused by an activated immune system in the patients.

Okuma et al. report a case of a 63-year old man suffering from hepatocellular carcinoma. After extended right hepatic lobectomy, a single lung metastasis and a single mediastinal lymph-node metastasis were found (106). As the first therapy option in the form of trans-catheter arterial embolization failed to work and carried the risk of spinal artery embolism, the patient received RT with a total dose of 60.75 Gy in 2.25 Gy fractions. Computed tomography scans showed significant reduction of the mediastinal lymph-node metastasis and spontaneous shrinking of the lung metastasis that was located outside of the irradiation field. No chemotherapy was given during the treatment and there has been no recurrence of either of the metastases during a 10-year follow-up period after RT.

There are in particular hints, that additional IT further enhances these effects. Golden et al. conducted a proof-of-principal trial (ClinicalTrials.gov, NCT02474186) using granulocyte-macrophage colony-stimulating factor (GM-CSF) as an effective stimulator of DC maturation (107). Forty-one patients with stable or progressing metastatic solid tumors who received single-agent CT or hormonal therapy were treated with concurrent RT (35 Gy total dose; 3.5 Gy/fractions for 2 weeks) to one metastatic site as well as daily subcutaneous injections of 125 μg/m^2^ GM-CSF for 2 weeks, starting at the second week of RT. This regiment was repeated targeting a second metastatic site. They found abscopal responses in 11 (26.8%, 95% CI 14.2–42.9) out of 41 patients. They, thus, conclude that a combinatory therapy approach with RT and GM-CSF is able to produce out-of-field responses in patients with metastatic solid tumors.

Grimaldi et al. also suggest a possible synergistic effect of IT and RT (108): they examined patients with advanced melanoma who were treated with ipilimumab, an immune checkpoint blockade monoclonal antagonist, followed by RT. Out of 21 patients, 13 (62%) received RT to treat metastases in the brain and 8 patients directed at extracranial sites, respectively. Thirteen patients (62%) showed local responses and among those, systemicl responses were observed in 11 patients (52%). Out of those 11 patients, 9 had partial responses (43%) and 2 had stable disease (10%). As distant effects were only observed in patients showing local responses, they suggest a connection of local responses to RT with distant effects.

Taken together, all these clinical data suggest a vital role of the immune system after RT. It would, however, be beneficial to know whether certain radiation regiments have a superior effect over others in terms of immune stimulation.

### Impact of Radiation Dose and Fractionation on Side Effects

Fu et al. investigated the late effects of a phase ILE/II dose escalation trial (RTOG 83-13) for hypofractionated RT (109). In that study, 479 patients with advanced head and neck cancer were randomly assigned to receive doses of either 67.2, 72, 76.8, or 81.6 Gy delivered at 1.2 Gy/fraction twice a day for 5 days/week. Patients were subclassified by the delivered doses and by the average daily interfraction interval of ≤4.5 or >4.5 h, respectively. Distribution of patients resulted in well-balanced treatment groups and the median follow-up was 1.71 years (0.24–9.6) for all patients and 6.12 years for 85 alive patients. No significant impact in the occurrence of late effects was observed; however, the incidence of late effects significantly differed with respect to the daily interfraction interval. Multivariant analysis showed that a daily interfraction interval of ≤4.5 h was the only significant independent prognosis for the development of grade 3+ or grade 4 late effects (*p* = 0.0167 and *p* = 0.0013). This study showed no evident dose–response relationship for the investigated doses; however, it did stress the importance of other factors in RT.

The RTOG 90-03 study (110, 111) examined three different radiation schemes with regard to local-regional tumor control (LRC) in comparison to standard fractionation for squamous cell cancers (SCC) of the head and neck. For that reason, patients with stage III or IV SCC were randomized to the four treatment arms: (a) standard fractionation scheme at 70 Gy delivered in 35 daily 2 Gy fractions over 7 weeks; (b) hyperfractionated protocol with a total dose of 81.6 Gy administered at 68 twice-daily 1.2 Gy doses for 7 weeks; (c) accelerated fractionation, continuous meaning 72 Gy in 42 1.8 Gy fractions during 6 weeks; (d) accelerated fractionation with split with 67.2 Gy at 42 1.6 Gy fractions within 6 weeks with a 2-week break after a dose of 38.4 Gy. At the first follow-up (111), patients who were treated with hyperfractionated protocol and continuous accelerated fractionation showed significant better LRCs (*p* = 0.045 and *p* = 0.050, respectively) than patients receiving standard fractionation scheme. Accelerated fractionation with split patients, on the other hand, showed similar outcome to those treated with standard fractionation scheme. However, all experimental RT schemes had significantly higher acute side effects in comparison to the standard fractionation protocol. For LRC at 5 years, only hyperfractionated protocol was different from standard fractionation scheme (110), hyperfractionated protocol improved the overall survival (OAS; hazard rate 0.81, *p* = 0.05). Any other side effects or toxicities did not differ significantly from standard fractionation scheme. However, in a comparison of 7-week treatments with 6-week treatments, accelerated fractionation appeared to increase grade 3, 4, or 5 toxicity at 5 years (*p* = 0.06). Even though after 5 years only hyperfractionated protocol significantly improved LRC and OAS for patients with locally advanced SCC without an increase in late toxicity, it was also shown that accelerated fractionation and shorter treatment durations enhance toxicity. These results stress the importance of long-term follow-ups with regard to judge LRC as well as side effects.

### Impact of Radiation Dose and Fractionation on Anti-Tumor Effects

As there are several studies comparing the effects of conventional RT and hyperfractionated or accelerated RT in patients suffering from HNSCC, with unclear results in regard to OAS, Bourhis et al. carried out a meta-analysis in order to answer that question: they acquired data from 15 trials of patients suffering from HNSCC and grouped them in three categories: hyperfractionated, accelerated, and accelerated with total dose reduction, with OAS as the end point (112). Median follow-up of the analysis was 6 years and a significant survival benefit with altered fractionation protocols was found (absolute benefit of 3.4% at 5 years with a hazard rate of 0.92, 95% CI 0.86–0.97; *p* = 0.003). Among the fractionation protocols, hyperfractionated RT posed as a significantly better therapeutic option than accelerated RT (8 vs. 2% with accelerated fractionation without total dose reduction and 1.7% with total dose reduction, respectively). Next to OAS, there was also a visible benefit with regard to LRC also in favor of altered fractionation protocols (6.4% at 5 years; *p* < 0.0001). The observed benefit was significantly higher in the youngest patients [HR 0.78 (0.65–0.94) for under 50-year-olds, 0.95 (0.83–1.09) for 51- to 60-year-olds, 0.92 (0.81–1.06) for 61- to 70-year-olds, and 1.08 (0.89–1.30) for over 70-year-olds; *p* = 0.007].

Norihisa et al. examined SABR for oligo metastatic lung tumors. In their study, they involved a total of 34 patients with the following primary involved organs: lung (*n* = 15), colorectum (*n* = 9), head and neck (*n* = 5), kidney (*n* = 3), breast (*n* = 1), and bone (*n* = 1) (74). They used 6 MV photon beams to deliver 48 Gy (*n* = 18) or 60 Gy (*n* = 16) with 12 Gy/fraction within 4–18 days (median: 12 days). OAS, local relapse-free rate and progression-free rate at 2 years was 84.3, 90.0, and 34.8%, respectively, with no progression observed in tumors irradiated with 60 Gy. Pulmonary toxicities were observed in four grade 2 cases (12%) and one grade 3 case (3%). As a result, 48 vs. 60 Gy showed no significant differences in survival rates, but differed in the local progression rate (*p* = 0.078). Thus, a dose escalation from 48 to 60 Gy resulted in an increased LCR without an increase in incidence or severity of pulmonary toxicity [13 (72%) and 2 (11%) at 48 Gy and 10 (63%) and 2 (13%) at 60 Gy, respectively] (74).

Guckenberger et al. carried out a pattern of care analyses in stage I NSCLC patients that, among other factors, also examined various fractionation schemes. They found that the most commonly used fractionation protocols were 3 × 12.5 Gy (prescribed to the 60 to 65% isodose line, *n* = 147) and 3 × 15 Gy (prescribed to the 65% isodose line, *n* = 107). Among all patients within this pattern of care study (*n* = 582) only six received >10 fractions. They came to the conclusion that SABR is a safe and effective treatment option in stage I NSCLC (113), further they found a fractionation scheme using 3 × 15 Gy to be the preferred treatment option that resulted in local tumor control rates of >90% (101).

The UK standardization of breast RT (START) trial (international standard randomized controlled trial ISRCTN59368779) compared a dose of 50 Gy in 25 fractions given over 5 weeks with 41.6 or 39 Gy in 13 fractions given over 5 weeks in women with completely excised invasive breast cancer (pT1-3a, pN0-1, M0) (114). The 5-year results suggested that lower total doses of RT that are administered in fewer, larger fractions are at least as safe and effective as standard RT schemes in women after primary surgery for early breast cancer. The 10-year follow-up (median follow-up 9.3 years; IQR 8.0–10.0) confirmed that hypofractionation, if given in appropriate doses, can be a safe and effective treatment option for patients with early breast cancer. The study further supports a 40 Gy in 15-fraction regiment that has already been adopted by most UK centers as the standard of care for women requiring adjuvant radiotherapy for invasive early breast cancer.

Running and future trials will now have to identify whether high-dose RT is superior to induce anti-tumor immune responses compared to classical fractionated RT or hyperfractionated RT and in particular how the outcome is when RT is combined with IT. A recent study showed that SABRand IMRT induce different plasmatic cytokine changes in NSCLC patients. This supports the hypothesis that RT regimes of dose schedules and techniques have different impacts on induction of anti-tumor immunity (115). Exemplary studies for current knowledge on that subject are summarized in Table 2.

**Table 2:** **The impact of fractionation regiments on local and systemic responses in selected clinical studies**.

	Tumor entity	Fractionation regiment	Additional therapy	Observed effects	Source
Out-of field responses	Hepatocellular carcinoma	60.75 Gy in 2.25 fractions	–	Shrinkage of out-of-field metastases in the lung and lymph node	(106)
	Stable or progressing metastatic solid tumors	35 Gy in 3.5 Gy/fraction for 2 weeks	Subcutaneous GM-CSF + CT/hormonal therapy	Out-of-field responses in 11 (26.8%, 95% CI 14.2–42.9) out of 41 patients	(107)
	Advanced melanoma	5 × 4 Gy 3 × 10 Gy 25 × 2 Gy 1 × 20/24 Gy	Ipilimumab 3 mg/kg i.v. every 3 weeks for four doses	Out-of-field responses in 11 (52%) patients	(108)

Side effects	Advanced head and neck cancer	67.2/72/76.8 Gy in two 1.2 Gy/fractions/day for 5 days/week	–	No evidence for a dose–response relationship Daily interfraction interval ≤4.5 h as only significant prognosis for the development of grade 3+ and 4 late effects (*p* = 0.0167 and *p* = 0.0013)	(109)
	HNSCC; stage II or IV	(a) 35 × 2 Gy (b) 68 × 1.2 Gy twice a day (c) 42 × 1.8 Gy (d) 42 × 1.6 Gy with a 2-week break at 38.4 Gy	–	(b) and (c) ↑ LRC than (a) (*p* = 0.045; *p* = 0.050) (a) and (d) similar outcome (b)–(d) ↑ acute side effects than in (a) (b) ↑ AS in comparison to (a) (HR 0.81; *p* = 0.05) Only (b) ↑ LRC significantly without an increase in late toxicity Accelerated fractionation used (c), (d) ↑ grade 3, 4, 5 toxicity at 5-year follow-up	(110, 111)

Anti-tumor effects	HNSCC	(a) Hyperfractionated (b) Accelerated (c) Accelerated with total dose reduction	–	Survival benefit with altered fractionation protocols (absolute benefit of 3.4% at 5 years, HR 0.92, 95% CI 0.86-0.97; *p* = 0.003) (a) ↑ therapeutic option than (b) and (c) (a) ↑ LRC in favor of altered fractionation protocols (6.4% at 5 years, *p* < 0.0001) Younger patients ↑ effects (≤50 years: HR 0.78 [0.65–0.94]; 51–60 years HR 0.95 [0.83–1.09]; 61–70 years HR 0.92 [0.81–1.06]: >70 HR 1.08 [0.89–1.30]; *p* = 0.007)	(112)
	Oligo metastatic lung tumors	(a) 48 Gy in 12 Gy fractions (b) 60 Gy in 12 Gy fractions	–	No difference in survival rates Dose escalation from 48 to 60 Gy ↑ LCR (*p* = 0.078)	(74)
	Invasive breast cancer	(a) 50 Gy in 25 fractions (b) 41.6/39 Gy in 13 fractions	-	5- and 10-year follow-up: hypofractionation in appropriate doses can be a safe and effective treatment option 40 Gy in 15 fractions adopted as standard of care by most UK centers	(114)
	NSCLC	(a) 52 Gy in eight fractions (SABR) (b) 60 Gy in 25 fractions (IMRT)	–	(a) ↓ of IL-10 and IL-17 plasma levels in between the first and last day of treatment (b) ↓ of IL-1, IL-1ra, IL-2, IL-12, FGF-2, MIP-1α, MIP-1β, TGF-α, TNF-α, VEGF plasma levels within the first 4 weeks of treatment	(115)
	Hepatic metastases	(a) 3 × 10 Gy (b) 5 × 10 Gy (c) 5 × 12 Gy	–	Statistically relevant differences in response rates at 60 vs. 50 Gy and 60 vs. 30 Gy (*p* = 0.03; *p* = 0.001); ↑ LC in 60 Gy cohort	(116)
	Colorectal liver metastases	(a) 1 × 18–30 Gy (b) 3 × 12 (c) 6 × 4	72% ≥ 1 CT regiment 42% ≥ 2 CT regiment before SABR	Dose-dependent LC: 18-month LC 84% for total doses ≥42 Gy and 43% for total doses <42 Gy Recommendation for a >90% LC 3-fraction regiment is ≥48 Gy	(117)

*↑, increase; ↓, decrease; Gy, Gray; GM-CSF, granulocyte-macrophage colony-stimulating factor; i.v., intravenously; HNSCC, head and neck squamous cell carcinoma; LRC, local-regional tumor control; OAS, overall survival; HR, hazard rate; NSCLC, non-small cell lung cancer; SABR, stereotactic ablative body radiotherapy; IMRT, intensity-modulated radio therapy; IL, interleukin; FGF-2, fibroblast growth factor-2; MIP, macrophage inflammatory protein; TGF, transforming growth factor; VEGF, vascular endothelial growth factor; LC, local control; CT, chemotherapy*.

## Outlook and Summary

Recent studies led to a paradigm shift that RT can also achieve an immunostimulatory effect either by directly influencing immune cells or through a modification of the tumor microenvironment (53, 68, 73, 74). Consequently, more and more emerging evidence points to the fact that the choice of RT dose and fraction plays a crucial role in cancer therapy and current RT concepts might need to be modified in order to achieve the best local and systemic therapy outcome (27, 63). Due to the technical advancements of today, RT can be administered in a more and more precise matter that is opening the door for new treatment strategies, such as SABR. The latter seem to have a beneficial outcome over classical RT fractionation in pre-clinical animal models (63, 84), even though *in vitro* model systems suggest an advantage for classical fractionated RT (79, 80). This discrepancy shows that model systems that complement one another are needed to get hints about the immunogenic potential of distinct radiation doses and fractionation schemes. It further raises the question if doses, which are used in the clinic, can be directly transferred onto pre-clinical models or if they need adjustments based on the BED in order to more closely mimic the processes in the patient. Clinical studies also show that there are many more factors, such as interfraction intervals (109) or the duration of the observation period (110, 111), that need to be taken into consideration as it is true for the genetic signature of the individual tumor. In order to identify the most immunogenic doses and RT protocols, either as a stand-alone therapy option or in combination with CT and/or IT, we first need to understand the basic mechanisms and conduct appropriate studies (118, 119). It is also important to keep in mind that there is significant variation between patients with regard to RT side effects. This might result in the administration of sub-maximal doses as current thresholds are set to fit the needs of those who are the most sensitive to radiation (120), but that also means that SABR might not be the optimal therapy option for all patients. In that matter, patients could benefit from a more personalized medicine. Thus, in order to achieve an optimized treatment outcome, it seems to be necessary to go back from bed to bench side first to be able to ultimately bring new insights back to the clinic to deliver the best possible therapy to the patients.

## Author Contributions

LD drafted and wrote the manuscript, and designed figures and one table. AD drafted and contributed to writing of the manuscript, and designed one table. JH drafted and contributed to writing the part about modern radiation techniques. Dr. BF contributed to drafting of the manuscript. Prof. RF contributed to writing the part about clinical applications of radiotherapy. Prof. UG drafted and wrote the manuscript.

## Conflict of Interest Statement

The authors declare that the research was conducted in the absence of any commercial or financial relationships that could be construed as a potential conflict of interest.
